# Effect of incorporating recycled *Ficusaw keotsang* Makino seeds on the physicochemical and sensory properties of soymilk and tofu

**DOI:** 10.1016/j.fochx.2025.103342

**Published:** 2025-12-02

**Authors:** Shu-Hua Chiang, Yung-Chung Chang, Chen-Ting Hsiao, Chih-Wei Chen

**Affiliations:** a Department of Health and Creative Vegetarian Science, Fo Guang University, Yilan 262307, Taiwan; b Department of Food Science, National Ilan University, Yilan 26047, Taiwan; c Bachelor Degree Program in Food Safety/Hygiene and Laboratory Sciences, Chang Jung Christian University, Tainan 71101, Taiwan

**Keywords:** Recycled *Ficus awkeotsang*, Makino seeds, Soymilk, Tofu, Texture, Sensory evaluation

## Abstract

This study examines the incorporation of recycled *Ficus awkeotsang* Makino seeds (RC-AWS), a by-product of jelly preparation, into soymilk and tofu to enhance their nutritional and functional properties. RC-AWS powder was added at varying concentrations (0 %, 0.5 %, 1 %, 1.5 %) and evaluated for antioxidant components, antioxidant activity, physicochemical properties, and sensory attributes. Results indicate that the total phenolic and flavonoid contents, as well as DPPH scavenging activity and reducing power, increased with the addition of RC-AWS. Tofu texture parameters and color were significantly affected, with a 0.5 % addition achieving optimal hardness and overall acceptability. Sensory evaluations show that soymilk and tofu containing 0.5–1.0 % RC-AWS were most acceptable. These findings highlight the potential of RC-AWS as a functional ingredient in plant-based foods, promoting sustainable reuse and product innovation, particularly for elderly nutrition.

## Highlights

• RC-AWS powder significantly enhances antioxidant activities in soymilk and tofu.
• A 0.5 % addition of RC-AWS improved the texture and consumer acceptability of tofu.
• RC-AWS provides sustainable valorization of traditional Taiwanese by-products.
• Thermal processing affects the retention of phenolic compounds in soy-based products.
• Sensory acceptance decreased when RC-AWS exceeded 0.5 % in tofu.

## 1 Introduction

The rising global burden of chronic diseases has increased interest in functional foods beyond basic nutrition (Li et al., 2022). Plant-based diets rich in soy and whole grains are associated with reduced mortality and disease prevention (Li et al., 2022). Soy products (e.g., soymilk, tofu) provide plant protein, isoflavones, polyunsaturated fatty acids, and minerals such as calcium and iron that support cardiovascular and bone health, alleviate menopausal symptoms, and help reduce body fat (Asif & Acharya, 2013; Qin et al., 2022). Tofu is suitable for vegetarians and for lactose-intolerant populations—up to 95 % in parts of Asia and Africa (Sethi et al., 2016; Storhaug et al., 2017)—and is appropriate for older adults, a rapidly growing demographic (*National Development Council*, 2022). Given the aging-related risks of malnutrition due to dental and chewing difficulties, there is a need for functional foods with enhanced nutrition and suitable texture (*National Health Administration*, 2018). At the same time, sustainability in food production has gained increased importance, particularly the reuse of agricultural by-products. One underutilized resource is the seed of *Ficus awkeotsang* Makino, commonly known as jelly fig or “aiyu.” Traditionally, these seeds are soaked and rubbed to release pectin, which forms a natural gel used in Taiwanese summer beverages (Li et al., 2005). However, the residual seeds—still rich in iron, fatty acids (notably linoleic and linolenic acids), and dietary fiber—are typically discarded (Chen et al., 2012; Chua et al., 2008). This practice represents a missed opportunity for both nutritional enhancement and circular resource utilization.

Tofu quality is commonly tuned with coagulants (calcium sulfate, magnesium chloride, or glucono-δ-lactone) and with texture-modifying additions such as carrageenan, konjac glucomannan, starches, inulin, and cereal/okara fibers. These strategies improve yield, water-holding capacity, and firmness but can also darken color, increase stickiness, or add cost. Direct fortification with polyphenols can raise antioxidant readouts but frequently introduces bitterness/astringency, browning, and protein–phenolic complexation that weakens soybean gels and lowers consumer acceptance. In contrast, we evaluate the direct use of recycled *Ficus awkeotsang* seed residue (RC-AWS)—a fiber-rich by-product that still carries phenolic constituents and iron. The fiber/acidic moieties can interact with Ca^2+^ and the soy protein network, potentially enhancing structure at low levels while tempering the off-flavor and color penalties associated with free phenolics. This approach also supports circular use of a regional by-product.

Incorporating RC-AWS powder into soy-based products, such as soymilk and tofu, offers not only enhanced nutritional value but also alignment with sustainable food system goals. Past research indicates that incorporating plant-based powders into tofu can enhance antioxidant content and modify texture without compromising consumer acceptability (Wang et al., 2020). Moreover, as a protein-rich, soft-textured product, tofu enhanced with fiber, iron, and bioactives from RC-AWS may be especially beneficial for older adults needing nutrient-dense, easy-to-chew options—supporting improved health outcomes and quality of life.

Despite the well-documented nutritional profile of aiyu seeds and the widespread use of soy products, few studies have explored integrating recycled aiyu seeds into mainstream food systems. Therefore, this study evaluates the effects of RC-AWS powder on the physicochemical, antioxidant, and sensory properties of soymilk and tofu. We employ a rigorous research methodology, including controlled experiments and sensory evaluations, to identify the optimal levels of RC-AWS. Furthermore, the study investigates changes in antioxidant content and functional properties while assessing texture and palatability, particularly for older adult consumers.

By combining sustainable resource use with functional product innovation, this research advances food system circularity and chronic disease prevention strategies—and holds potential to revolutionize the food industry. Furthermore, valorizing aiyu seeds could unlock new market opportunities and boost the economic value of traditional Taiwanese agricultural products, benefiting both the local economy and the global food market.

## 2 Materials and methods

### 2.1 Sample materials and preparation

#### 2.1.1 Source and pre-treatment of RC *Ficus awkeotsang* Makino seeds

The recycled *Ficus awkeotsang* Makino seeds (RC-AWS) used in this study were obtained from the Lioujia campus of the Industrial Technology Research Institute in the Southern Region of Taiwan. These seeds were harvested initially from Aiyu Village in Taoyuan District, Kaohsiung, and were recovered after the traditional aqueous extraction of Aiyu jelly. Following extraction, the residual seeds were vacuum-dried (at 50 °C under ≤10 kPa (≤100 mbar) for 16–18 h) and stored under dry conditions for further use.

#### 2.1.2 Preparing RC-AWS powder

The dried RC-AWS was ground into a fine powder using a high-speed grinder. The powder was sieved sequentially using 40-mesh and 60-mesh stainless steel sieves to ensure uniform particle size. The screened powder was packed in light-protective zip-lock polyethylene bags and stored at 4 °C. This temperature was carefully chosen to prevent oxidation and moisture absorption before subsequent experiments.

#### 2.1.3 Ultrasound-assisted extraction of RC-AWS powder

This study used ultrasound-assisted solvent extraction to obtain bioactive components from RC-AWS powder. The procedure was adapted from the methods of Ma et al. (2019) and Sharma et al. (2020) with modifications. Precisely weighed RC-AWS powder was mixed with extraction solvents—distilled water, 70 % ethanol, and 95 % ethanol—at two solid-to-liquid ratios (1:100 and 1:200, *w*/*v*). The mixtures were subjected to ultrasonic extraction using a 40 kHz ultrasonic bath at 50 °C for 30 min. Following extraction, the mixtures were centrifuged at 3000 ×*g* for 15 min to separate the supernatant. The resulting extracts were vacuum-filtered to remove insoluble residues and adjusted to a final volume of 100 mL. Extracts were stored in airtight containers at 4 °C until further analysis of antioxidant activity and phytochemical composition.

### 2.2 Preparing soymilk containing RC-AWS powder

Soymilk preparation was carried out meticulously, following the method of Yu et al. (2021), with slight modifications. Dried soybeans (100 g) were rinsed under running water and soaked in 10 times their weight of water (1000 mL) at room temperature for seven hours. After soaking, the beans and soak water were transferred into a high-speed blender and homogenized at 12000 rpm for 1 min to obtain a slurry. The slurry was filtered through a soybean milk cloth filter (130–140 mesh) to separate the raw soymilk from the okara (soy pulp), ensuring soymilk purity. RC-AWS powder was added to the raw soymilk at four concentrations (0 %, 0.5 %, 1.0 %, and 1.5 % *w*/w relative to soymilk weight) and mixed thoroughly. The fortified soymilk was boiled in a stainless-steel pot on a portable gas stove. It was maintained at 95 °C for 5 min to ensure proper cooking. After cooling, the soymilk was filtered again through a cotton-based soymilk bag to remove the insoluble RC-AWS residue. The final RC-AWS fortified soymilk was divided into two portions: one was filled into sterilized glass bottles and stored at 4 °C, while the other was stored in zip-lock bags at −20 °C for freeze-drying and analysis.

### 2.3 Extracting bioactive compounds from RC-AWS fortified soymilk

The extraction of antioxidant components from RC-AWS fortified soymilk was performed using the modified method of Chen et al. (2013). Approximately 1 g of freeze-dried RC-AWS-fortified soymilk powder was weighed and transferred into a 50 mL centrifuge tube containing 25 mL of 70 % ethanol. The sample was placed on a rotary shaker and agitated at 200 rpm at 50 °C for two hours to ensure thorough extraction. After extraction, the tubes were centrifuged at 8800 ×*g* for 10 min at 4 °C. The resulting supernatant—containing the extracted bioactive compounds—was carefully collected and stored at −20 °C to preserve the integrity of the compounds until analysis of their antioxidant properties and activity.

### 2.4 Preparing tofu fortified with RC-AWS powder

The tofu manufacturing process was adapted from Cai et al. (1997) with modifications. Soybeans (300 g) were rinsed and soaked in water at a 1:7 (*w*/w) bean-to-water ratio for seven hours. The soaked beans and water were homogenized in a high-speed blender for 1 min. The resulting slurry was filtered through a 130–140 mesh soybean cloth filter to obtain raw soymilk. RC-AWS powder was incorporated into the soymilk at concentrations of 0 %, 0.5 %, 1.0 %, and 1.5 % (*w*/w). The mixture was transferred to a stainless-steel pot and cooked over medium heat, stirring constantly for 10 min until it reached boiling temperature. Heating continued at 95 °C for 5 min to ensure complete gelatinization. When the soymilk cooled to around 85 °C, it was quickly poured into a stainless-steel container with 50 mL of gypsum coagulant solution. The calcium sulfate was prepared at concentrations equivalent to 0.2 %, 0.3 %, or 0.4 % of the soymilk weight. The mixture was gently stirred and allowed to stand undisturbed for 20 min to form curds. The curds were carefully ladled into square wooden molds lined with cotton cloth. The sides of the cloth were folded over the surface and covered with a wooden press lid. Pressing was performed in three stages to shape and dehydrate the tofu: 21.8 g/cm^2^ for 10 min, 43.6 g/cm^2^ for 10 min, and 65.4 g/cm^2^ for 15 min. After pressing, the former tofu was demolded, weighed, and stored at 4 °C for further analyses.

### 2.5 Determining the proximate compositions

Moisture, ash, crude fat, and crude fiber contents were determined using the methods of the Association of Official Analytical Chemists (A.O.A.C., 2022), specifically methods 934.01, 942.05, 920.39, and 985.29, respectively. Crude protein content (N × 6.25) was determined using the AOAC Kjeldahl method 984.13, which measures a substance's nitrogen content to calculate the protein content. Total solids content was calculated as 100 % minus the moisture content (%). Carbohydrate content was obtained by subtracting the crude ash, crude protein, and crude fat contents measured in the above samples. The calculation formula is as follows:

$$\text{Carbohydrate}\;\left(\%\right)=100\;\left(\%\right)-\text{crude}\;\mathrm{fat}\;\left(\%\right)-\text{crude protein}\;\left(\%\right)-\text{crude}\;\mathrm{ash}\;\left(\%\right)$$

### 2.6 Determining Total polyphenol content (TPC)

The TPC of the RC-AWS ultrasound-assisted extract and RC-AWS-fortified soymilk extract was determined using a modified Folin–Ciocalteu spectrophotometric method based on Padhi et al. (2017). Briefly, 25 μL of either gallic acid standard solution (0.0–0.2 mg/mL) or test samples were added to each well of a 96-well microplate. Next, 125 μL of 0.2 M Folin–Ciocalteu reagent was added, followed by 100 μL of 1 N sodium carbonate (Na₂CO₃) solution. The contents of each well were gently mixed by pipetting, and the plate was covered with aluminum foil to protect it from light. The mixture was incubated at room temperature for 45 min under dark conditions. Following incubation, absorbance was measured at 765 nm using a microplate spectrophotometer. Gallic acid was used as the standard to establish a calibration curve (R^2^ > 0.995). The results were expressed as milligrams of gallic acid equivalents per gram of dry weight (mg GAE/g DW).

### 2.7 Determining Total flavonoid content (TFC)

The TFC of the RC-AWS ultrasound-assisted extract and RC-AWS-fortified soymilk extract was determined using a comprehensive colorimetric method based on Dutra et al. (2019). In each well of a 96-well microplate, 100 μL of either quercetin (Q) standard solution (ranging from 0 to 0.15 mg/mL) or samples was added. Next, 100 μL of 2 % aluminum chloride hexahydrate (AlCl₃·6H₂O) in ethanol was added to initiate complexation with flavonoids. The microplate was covered with aluminum foil to avoid light exposure and allowed to react at room temperature for 10 min under dark conditions. Following incubation, the foil was removed, and absorbance was measured at 430 nm using a microplate reader(ELISA (BioTEK® Instruments, Inc., U.S.A)). A standard curve was constructed using quercetin as the reference compound. The results are expressed as milligrams of quercetin equivalents per gram of dry weight (mg QE/g DW).

### 2.8 Determining antioxidant activity

#### 2.8.1 DPPH radical scavenging activity

The radical scavenging activity of the samples was determined using the DPPH assay described by Amarowicz et al. (2010), with minor modifications. A 96-well microplate was used, and 50 μL of either the sample solution or Trolox standard (0.00–0.15 mg/mL) was pipetted into each well. Next, 250 μL of freshly prepared 0.25 mM DPPH solution in methanol was added to each well. The microplate was covered with aluminum foil and incubated in the dark at room temperature for 30 min. Following incubation, the foil was removed, and absorbance was measured at 517 nm using a microplate reader. A calibration curve was constructed based on the absorbance of Trolox standards. The results are expressed as milligrams of Trolox equivalents per gram of dry weight (mg TE/g DW). All measurements were performed in triplicate.

#### 2.8.2 Reducing power

The reducing power of the samples was assessed using the method described by Sun et al. (2017) with slight modifications. In a 1.5 mL microcentrifuge tube, 75 μL of either the sample or an ascorbic acid standard solution (0.00–0.10 mg/mL) was mixed with 75 μL of sodium phosphate buffer (pH 6.6) and 75 μL of 1 % potassium ferricyanide. The mixture was incubated in a 50 °C water bath for 20 min. After cooling to room temperature, 300 μL of distilled water, 75 μL of 10 % trichloroacetic acid, and 300 μL of 0.1 % ferric chloride were added. The mixture was left to react in the dark for 15 min. A 210 μL aliquot of each reaction mixture was transferred to a 96-well microplate, and absorbance was recorded at 700 nm using a microplate reader. The reducing power was quantified using a standard curve generated with ascorbic acid. The results are expressed as milligrams of ascorbic acid equivalents per gram of dry weight (mg AAE/g DW).

### 2.9 Physicochemical properties analysis

#### 2.9.1 Color measurement

The color attributes of RC-AWS tofu were measured using a colorimeter (CR-400/410 KONICA MINOLTA, Japan) according to standard procedures. Tofu samples were cut into uniform blocks and placed on a flat, non-reflective surface for analysis. Color measurements were recorded in the CIE Lab* color space, where L* indicates lightness, a* represents red-green intensity, and b* indicates yellow–blue intensity.

#### 2.9.2 Texture profile analysis (TPA)

The tofu's textural characteristics were evaluated based on the method of Tang (2007), which employed a texture analyzer (Texture Analyzer, TA.XT plus, Stable Micro Systems, Godalming, Surrey, UK) equipped with a 3 cm diameter cylindrical probe (adaptor SMS P/75). Before testing, tofu samples were removed from refrigeration and allowed to equilibrate to room temperature. Each sample was cut into uniform cubes measuring 2 × 2 × 2 cm^3^. A double compression cycle was applied to simulate mastication. The probe compressed the to futo 75 % of its original height, then returned to its starting position. Test parameters included a pre-test speed of 1.5 mm/s and a compression load of 5 kg. Six textural attributes were evaluated: hardness, springiness, cohesiveness, adhesiveness, chewiness, and resilience. For each formulation, 15 measurements were recorded, and mean values were calculated.

#### 2.9.3 Determining Iron content

The iron content in the RC-AWS powder and RC-AWS tofu was determined based on the Taiwan National Standard (CNS 12869 N6231). Iron content (μg/g) was calculated using the following formula:

$$\text{Iron content}\;\left(\mathrm{\mu g}/g\right)=C\times \frac{100}{V}\times \frac{100}{W.}$$

### 2.10 Sensory analysis: Consumer hedonic test

The sensory evaluation protocol was reviewed and approved by the Institutional Review Board of National Yang-Ming Chiao Tung University Hospital (IRB No.: 2025E001). All participants were fully informed about the study's objectives, procedures, and potential risks, and they provided written informed consent prior to participation. All personal data was anonymized to ensure privacy and confidentiality. The sensory evaluation was conducted in the food sensory evaluation classroom of Yilan University in Yilan, Taiwan. A total of 50 students and 35 teachers from Yilan University were recruited to test soy milk and tofu, respectively. We used a 9-point hedonic scale for sensory evaluation. Prior to the test, samples were coded with three digits using a random number table and stored in a refrigerator at 4 °C for later use. Panelists were asked to evaluate the samples in the order of the numbers on the evaluation sheet. The evaluation sheet was divided into two parts. The first part used a 9-point method to evaluate consumers' perception of the sample's appearance, aroma, taste, flavor, willingness to purchase, and overall acceptance. The tofu evaluation sheet included descriptions of appearance, flavor, and taste.

### 2.11 Statistical analysis

All experimental data were expressed as mean ± standard deviation (SD). This study performed statistical analyses using SAS statistical software (version 9.0, SAS Institute Inc., Cary, NC, U.S.A). We used a one-way analysis of variance (ANOVA) to evaluate the significance of differences among treatment groups, followed by Duncan's multiple range test for post hoc comparisons. A significance level of *P* < 0.05 was considered statistically significant. Correspondence analysis (CA) was performed using the check-all-that-apply (CATA) method, where 1/0 was used to indicate whether the panelist selected the sample. The samples (columns) and the number of descriptor selections (columns) were tabulated in a column-by-column format to perform a Cochran's Q test, which assessed whether there were significant differences in descriptors between samples (*p* < 0.0001) and evaluated correlations between samples and descriptors.

## 3 Results and discussion

### 3.1 Proximate composition analysis

The proximate composition of the RC-AWS was analyzed after drying at 105 °C for 24 h, followed by grinding and sieving through a 40-mesh screen. The RC-AWS powder had a moisture content of 5.54 ± 0.28 %, total carbohydrates of 77.34 ± 1.10 %, crude protein of 9.23 ± 0.25 %, crude fat of 2.63 ± 0.30 %, ash content of 5.27 ± 1.18 %, crude fiber of 23.00 ± 3.45 %, and iron content of 0.28 mg/g (Table 1). Compared to the nutritional profile of raw Aiyu seeds published by the Taiwan Food and Drug Administration (TFDA, 2017)—moisture 7.8 %, total carbohydrates 63.7 %, crude protein 12.2 %, crude fat 12.5 %, ash 3.8 %, and iron 0.08 mg/g—the RC-AWS demonstrated a substantial reduction in crude fat and crude protein content. This was likely due to nutrient leaching during the jelly extraction process. The pectic substances and water-soluble proteins may have been partially dissolved and removed, contributing to the observed decline in these macronutrients. Notably, the RC-AWS had significantly higher levels of total carbohydrates and ash content. The apparent increase could be attributed to a relative concentration effect resulting from the loss of lipids and proteins. This could lead to a higher percentage of residual carbohydrates, including insoluble polysaccharides and non-extractable carbohydrates. Increased ash content and, subsequently, iron content (0.28 mg/g vs. 0.08 mg/g in raw seeds) may also reflect the enrichment of mineral constituents after water-extractable compounds have been removed. Notably, the crude fiber content remained relatively high at 23.00 ± 3.45 %, indicating that a significant portion of the structural polysaccharides—including cellulose and residual pectin—was retained post-extraction. This fiber-rich profile suggests that RC-AWS could serve as a functional dietary fiber source in food applications, particularly soy-based products with improved potential for gastrointestinal and cardiovascular health.

**Table 1:** Proximate composition of *Ficus awkeotsang* Makino seed.

Composition	RC-AWS (%)	TFDA (%)	Ref. (%)
Moisture	5.54 ± 0.28	7.8	10.3
Total solids	94.46 ± 0.28	92.2	89.7
Crude ash	5.27 ± 1.18	3.8	3.7
Crude fat	2.63 ± 0.30	12.5	10.2
Crude protein	9.23 ± 0.25	12.2	10.5
Carbohydrate	77.34 ± 1.10	63.7	65.3
Crude fiber	23.00 ± 3.45	51.6	16.1
Iron(mg/g)	0.28 ± 0.00	0.1	–

Values are presented as mean ± standard deviation., *n* = 3.

-: No data.

AWS: *Ficus awkeotsang Makino* seed.

TFDA: Taiwan Food and Drug Administration.

Ref.: Commercially available ayi.

### 3.2 Total antioxidant components and antioxidant ability of RC-AWS extracts

**Fig. 1:**
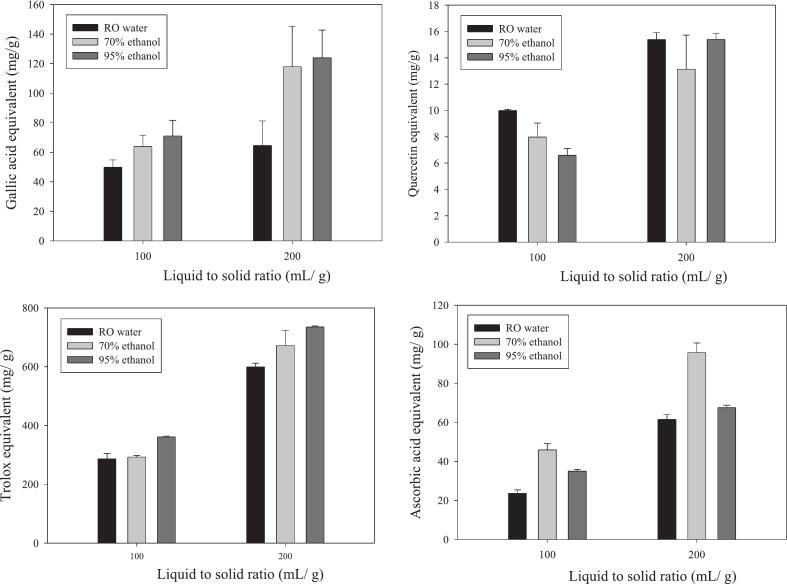
Effects of ethanol concentration and liquid to solid ratio on the total phenolic content (A), flavonoid content (B), DPPH radical scavenging activity (C), and reducing power (D) in the extract of recycled Ficus awkeotsang Makino seed powder.

The antioxidant components of the RC-AWS extracts, including total phenolic content (TPC) and total flavonoid content (TFC), were significantly affected by the choice of extraction solvent and liquid-to-solid ratio. As polyphenol and flavonoid extraction efficiency often varies depending on plant species, tissue type, and solvent polarity, selecting a suitable solvent system was essential for maximizing yield. This study tested water, 70 % ethanol, and 95 % ethanol in combination with liquid-to-solid ratios of 1:100 and 1:200 (mL/g) under ultrasonic-assisted extraction conditions (40 kHz, 50 °C, 30 min). The results are presented in Fig. 1. For TPC (Fig. 1A), the data show that increasing the liquid-to-solid ratio from 100 to 200 substantially enhanced extraction efficiency, regardless of the solvent used. Among all treatments, 95 % ethanol at a 1:200 ratio yielded the highest TPC (123.9 mg GAE/g DW), followed by 70 % ethanol (118.0 mg GAE/g DW) and water (64.6 mg GAE/g DW). The superiority of 95 % ethanol aligns with past research indicating that high-polarity protic solvents are most effective for extracting phenolics (Rezaie et al., 2015). Compared to water, 95 % ethanol achieved nearly 1.9 times the TPC and was 1.05 times more effective than 70 % ethanol. These results suggest that ethanol, a GRAS (Generally Recognized as Safe) solvent, is suitable and efficient for extracting phenolics from RC-AWS. These findings differ from Fernández-Agulló et al. (2013), who reported better polyphenol recovery using 50 % aqueous ethanol. The discrepancy may be attributed to the ultrasonic extraction method used in this study or the unique physicochemical nature of RC-AWS, which differs from that of typical plant tissues. Moreover, some studies have reported that medicinal plants, e.g., Scutellaria baicalensis and Cuscuta chinensis, yielded higher phenolic contents when extracted with ethanol. In contrast, others, e.g., *Codonopsis pilosula* and *Curcuma longa*, performed better with water extraction, indicating that the effect of the solvent is species- and matrix-dependent. Regarding TFC (Fig. 1B), extraction outcomes varied more intricately with solvent polarity. At a 1:100 ratio, TFC decreased as ethanol concentration increased, yielding the highest content with water (10.0 mg QE/g DW), followed by 70 % ethanol (8.0 mg QE/g DW), and 95 % ethanol (6.6 mg QE/g DW). However, when the liquid-to-solid ratio was increased to 1:200, 95 % ethanol demonstrated a substantial improvement (15.37 mg QE/g DW), comparable to water (15.40 mg QE/g DW), while 70 % ethanol remained less effective (13.1 mg QE/g DW). These findings suggest flavonoid extraction from RC-AWS may be more solvent-sensitive and influenced by solubility dynamics. Similar variation was reported by Ojeda et al. (2022), who noted that optimal ethanol concentrations for extracting polyphenols from mango tissues ranged from 25 % to 49 %, depending on the plant part (peel, pulp, or seed). The results of the present study also align with Zhang et al. (2011), who found that the flavonoid yield of different herbs responded variably to water versus ethanol extractions. Overall, this study demonstrates that ultrasonic-assisted extraction with 95 % ethanol at a 1:200 ratio yields the highest amount of phenolic compounds from RC-AWS. For flavonoids, both water and 95 % ethanol were comparably effective at higher extraction ratios. These results suggest that matrix hydration and solvent accessibility play key roles in the release of these bioactives. This insight informs optimal extraction strategies to valorize RC-AWS as a functional food ingredient enriched with antioxidants. The antioxidant capacity of RC-AWS extracts was assessed via DPPH radical scavenging activity and ferric reducing antioxidant power (FRAP), with results expressed as Trolox equivalent (TE) and ascorbic acid equivalent (Vit. C), respectively. The effects of extraction solvent (water, 70 % ethanol, 95 % ethanol) and liquid-to-solid ratio (100 and 200 mL/g) were compared (Fig. 1C and D). Fig. 1C shows that DPPH radical scavenging activity was significantly enhanced at a liquid-to-solid ratio of 200, regardless of the solvent used. Notably, 70 % ethanol extraction at this ratio had a 2.2-fold increase in TE value (671.8 mg TE/g DW) compared to ratio 100 (292.8 mg TE/g DW). Water and 95 % ethanol had approximately 2.0-fold increases (599.2/286.4 and 734.8/360.9 mg TE/g DW, respectively). Among solvents at a 1:200 ratio, 95 % ethanol had the highest DPPH activity, followed by 70 % ethanol and water. Fig. 1D shows that FRAP was most pronounced with 70 % ethanol at both extraction ratios. At a ratio of 200, the 70 % ethanol extract had the highest reducing power (95.8 mg Vit. C/g DW), outperforming the 95 % ethanol extract (67.5 mg Vit. C/g DW) and the water extract (61.5 mg Vit. C/g DW). The same trend was observed at a ratio of 100, with 70 % ethanol (45.9 mg Vit. C/g DW) exceeding 95 % ethanol and water by 1.3 and 1.9 times, respectively. Notably, there was a discrepancy between the content of antioxidant components and their antioxidant activity. For instance, although water-extracted samples showed the highest flavonoid content (Fig. 1B), their antioxidant capacity was the lowest. Conversely, 70 % ethanol, despite yielding less total phenolics and flavonoids than 95 % ethanol, exhibited the highest reducing power. This finding suggests the presence of other antioxidant compounds in RC-AWS that were more effectively extracted with 70 % ethanol. Previous studies have also reported inconsistent correlations between antioxidant activity and phenolic or flavonoid content (Zhang et al., 2011). For instance, high antioxidant activity was observed in *Eucommia ulmoides* and Gaultheria leucocarpa, despite their low phenolic content, indicating the involvement of other bioactive constituents beyond phenolics and flavonoids. Overall, both antioxidant assays support the conclusion that a higher liquid-to-solid ratio (200 mL/g) enhances extraction efficacy. Among solvents, 70 % ethanol had the highest reducing power, while 95 % ethanol provided superior DPPH radical scavenging activity. Thus, 70 % ethanol with a 1:200 ratio was identified as the optimal extraction condition for maximizing the antioxidant activity of RC-AWS.

### 3.3 Antioxidant components and activities of RC-AWS fortified soymilk

**Fig. 2:**
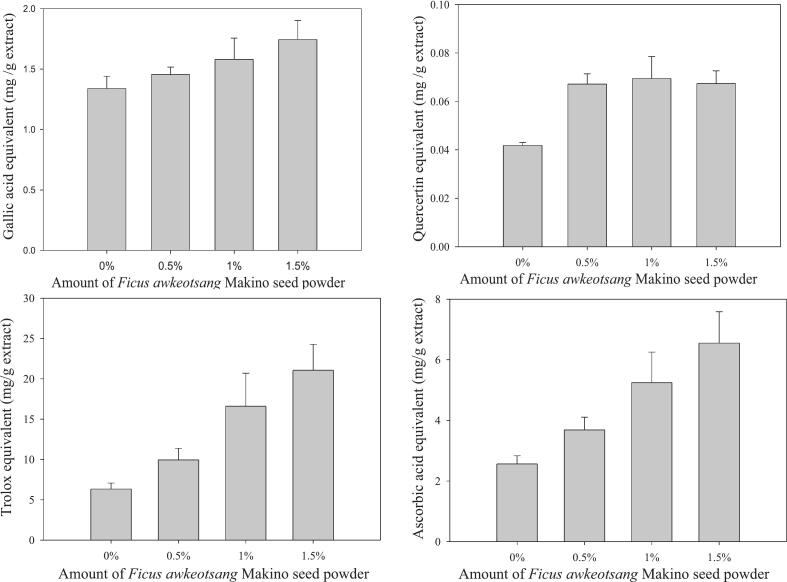
Total phenolic content (A) and flavonoid content (B) DPPH radical scavenging activity (C), and reducing power (D) in the 70 % ethanolic extracts of soybean milk prepared by adding recycled Ficus awkeotsang Makino seed powder.

To evaluate the effects of incorporating RC-AWS powder on the antioxidant properties of soymilk, freeze-dried samples containing 0 %, 0.5 %, 1.0 %, and 1.5 % RC-AWS were extracted using 70 % ethanol. The total phenolic content (TPC) and total flavonoid content (TFC) of the extracts are presented in Fig. 2A and B, respectively. Fig. 2A shows a linear increase in TPC with rising levels of RC-AWS fortification, from 1.34 mg GAE/g DW in the control (0 %) to 1.75 mg GAE/g DW at 1.5 % addition. Fig. 2B reveals an upward trend in TFC, from 0.042 mg QE/g DW at 0 % to 0.069 mg QE/g DW at 1 %. However, a plateau was observed at 1.5 % (0.067 mg QE/g DW), suggesting saturation or degradation at higher addition levels. However, TPC and TFC values were substantially lower in the fortified soymilk extracts than in the direct RC-AWS extracts, likely due to thermal degradation during soymilk preparation at 95 °C. Vuong, Golding, Nguyen and Roach, 2010, Vuong et al., 2013 demonstrated that polyphenol extraction efficiency initially increases with temperature due to enhanced solvent diffusion and solubilization. However, excessive heat (>80 °C) leads to the degradation of catechin and phenolic compounds, ultimately reducing their antioxidant capacity. Measured by DPPH radical scavenging and reducing power assays, antioxidant activity is illustrated in Fig. 2C and D, respectively. As RC-AWS content increased, both antioxidant indicators improved. Specifically, DPPH activity rose from 6.33 ± 0.75 mg TE/g DW (0 %) to 21.05 ± 3.22 mg TE/g DW (1.5 %), while reducing power increased from 2.56 ± 0.27 mg Vit. C/g DW to 6.55 ± 1.03 mg Vit. C/g DW. The upward trend in antioxidant activity mirrored the TPC pattern, supporting a strong correlation between phenolic content and antioxidant capacity. Moreover, a 0.5 % increment in RC-AWS powder resulted in approximately a 60 % increase in DPPH activity and a 50 % enhancement in reducing power. These findings suggest that incorporating RC-AWS into soymilk adds nutritional value and enhances its antioxidant potential despite some losses due to thermal processing.

### 3.4 Proximate composition of tofu fortified with RC-AWS powder

Tofu samples were prepared by incorporating 0 %, 0.5 %, 1 %, and 1.5 % RC-AWS powder into raw soymilk (soybean-to-water ratio 1:7, *w*/w). The coagulation was conducted using calcium sulfate (CaSO₄) at concentrations of 0.4 %, 0.3 %, or 0.2 % (based on soymilk weight). After heating the soymilk to 85 °C, it was rapidly mixed with the coagulant, allowed to stand, and coagulated into curds. The curds were molded and pressed to yield firm tofu. Table 2 presents the proximate composition of RC-AWS-fortified tofu. Among samples prepared with 0.4 % CaSO₄, increasing RC-AWS addition from 0 % to 1.5 % consistently decreased moisture content (82.1 % to 77.6 %), crude protein (49.2 % to 45.1 %, dry basis), and ash (7.2 % to 5.2 %). Conversely, total carbohydrates (from 18.1 % to 23.7 %) and crude fiber (from 1.1 % to 8.4 %) increased with higher RC-AWS levels, while crude fat content remained nearly constant (25.5 % to 26.1 %). These trends reflect the inherent composition of the RC-AWS powder, which is primarily composed of carbohydrates (77.4 %), including a high proportion of crude fiber (23.0 %), along with modest levels of protein (9.2 %), ash (5.3 %), and low moisture (5.5 %). Therefore, its inclusion naturally enhances the tofu's carbohydrate and fiber content while diluting protein and ash content. The observed reduction in protein and moisture levels with increasing RC-AWS can also be attributed to the competitive binding of calcium ions. Pectic polysaccharides and negatively charged carboxylate groups in the RC-AWS matrix may bind Ca^2+^, reducing their availability to cross-link with soy proteins and weakening curd formation. This phenomenon is consistent with the findings of Abd Karim et al. (2022), who demonstrated that both proteins and polysaccharides can independently bind Ca^2+^ and contribute to forming a gel network. Therefore, excessive RC-AWS addition may lead to either weak gelation or overcoagulation with increased syneresis, resulting in protein loss and reduced water retention. Furthermore, varying the calcium sulfate concentration also affected the tofu's composition. At a fixed RC-AWS level (e.g., 0.5 %), reducing the coagulant concentration from 0.4 % to 0.2 % resulted in a decrease in ash content (6.2 % to 5.0 %) while slightly improving moisture and protein retention. This trend aligns with the coagulation balance theory proposed by Kao et al. (2003), in which insufficient calcium results in weak gels prone to protein and water loss, while excessive calcium leads to overly compact structures and syneresis. Hence, moderate coagulant levels (e.g., 0.3 %) may offer optimal protein retention and yield. Based on the above, incorporating RC-AWS powder into tofu formulations significantly enhances the dietary fiber and carbohydrate content. However, this must be carefully balanced with the coagulant dosage to maintain desirable moisture and protein levels in the final product.

**Table 2:** Proximate composition of tofu prepared by adding recycled *Ficus awkeotsang* Makino seed powder and gypsum.

		Chemical composition (%)					
		fresh weight.	dry weight.				
Gypsum (%)	RC-AWS (%)	Moisture	Crude protein	Crude fat	Ash	Crude Fiber	Carbohydrate
0.4	0	82.10 ± 0.19^a^	49.24 ± 1.11^a^	25.51 ± 0.79^ab^	7.17 ± 0.23^a^	1.05	18.08 ± 2.02^c^
	0.5	80.28 ± 0.19^c^	45.93 ± 0.63^cde^	26.43 ± 0.26^a^	6.20 ± 0.28^b^	3.04	21.45 ± 0.98^ab^
	1	78.94 ± 0.11^d^	46.85 ± 0.69^bcd^	25.12 ± 0.54^b^	5.19 ± 0.09^cd^	6.30	23.09 ± 0.60^ab^
	1.5	77.57 ± 0.26^e^	45.06 ± 0.31^e^	26.07 ± 0.74^ab^	5.22 ± 0.23^cd^	8.39	23.65 ± 0.68^a^
0.3	0.5	81.19 ± 0.25^d^	47.36 ± 0.90^bc^	25.84 ± 0.54^ab^	5.37 ± 0.09^c^	3.44	21.43 ± 1.21^b^
0.2	0.5	81.23 ± 0.25^b^	47.98 ± 0.98^ab^	25.19 ± 0.24^b^	4.98 ± 0.06^d^	3.44	21.85 ± 0.95^ab^

Data are expressed as mean ± standard deviation (n = 3).

Data with different letters within the same column are significantly different (p < 0.05).

RC-AWS: Recycled *Ficus awkeotsang* Makino seed powder.

### 3.5 Color analysis of tofu fortified with RC-AWS powder

The color parameters (L*, a*, b*) of tofu samples containing 0–1.5 % RC-AWS powder and coagulated with 0.2–0.4 % calcium sulfate were evaluated using a colorimeter. The results are presented in Table 3. Under a fixed coagulant concentration of 0.4 %, the control tofu (0 % RC-AWS) had the highest lightness (L* = 83.18 ± 0.16) and yellowness (b* = 14.29 ± 0.51), along with the lowest redness (a* = 0.12 ± 0.11). In contrast, tofu with 1.5 % RC-AWS exhibited a markedly darker and redder appearance, with the lowest lightness (L* = 66.24 ± 0.54), the lowest yellowness (b* = 9.76 ± 0.48), and the highest redness (a* = 5.37 ± 0.22). These results show that increasing levels of RC-AWS powder progressively decrease brightness and yellowness while simultaneously increasing redness. The visual darkening and reddish tint of the tofu is likely attributable to the inherent color characteristics of the RC-AWS powder, which intensifies as more is incorporated. Thus, RC-AWS powder adds dietary fiber and bioactive compounds while significantly altering the aesthetic appearance of tofu, making it appear darker and redder at higher inclusion levels. In contrast, varying the calcium sulfate concentration (0.2–0.4 %) had a minimal impact on tofu color. For example, tofu samples containing 0.5 % RC-AWS across coagulant levels showed minimal differences in color parameters, with lightness ranging from 74.04 to 75.41, redness from 2.05 to 2.64, and yellowness from 9.11 to 9.92. These minor variations suggest that calcium sulfate, being colorless, does not significantly influence tofu color at the tested concentrations. This finding is supported by Kamizake et al. (2016), who noted that tofu inherits its color from the soymilk and added ingredients rather than the coagulant itself. Similarly, Jayasena et al. (2014) reported that various types of coagulants (e.g., calcium sulfate, magnesium chloride, calcium acetate) do not significantly affect tofu color unless the coagulants contain pigmented impurities. Therefore, the primary determinant of tofu color in this study is the amount of RC-AWS powder added to the soymilk before coagulation.

**Table 3:** The color parametersof tofu prepared by adding recycled *Ficus awkeotsang* Makino seed powder and gypsum.

Gypsum(%)	RC-AWS(%)	L*	a*	b*
0.4	0.0	83.18 ± 0.16^a^	0.12 ± 0.11^f^	14.29 ± 0.51^a^
	0.5	75.41 ± 0.28^b^	2.09 ± 0.04^e^	9.89 ± 0.24^b^
	1.0	71.79 ± 0.11^d^	3.64 ± 0.12^c^	9.05 ± 0.25^d^
	1.5	66.24 ± 0.54^f^	5.37 ± 0.22^a^	9.76 ± 0.48^bc^
0.3	0.5	74.64 ± 0.83^bc^	2.05 ± 0.30^e^	9.11 ± 0.47^cd^
	1.0	68.48 ± 0.96^e^	4.07 ± 0.34^b^	9.78 ± 0.07^bc^
0.2	0.5	74.04 ± 1.28^c^	2.64 ± 0.07^d^	9.92 ± 0.41^b^

Data are expressed as mean ± standard deviation (n = 3).

Data with different letters within the same column are significantly different (*p* < 0.05).

RC-AWS: Recycled *Ficus awkeotsang* Makino seed powder Data are expressed as mean ± standard deviation (*n* = 3).

### 3.6 Textural properties of tofu fortified with RC-AWS powder

**Fig. 3:**
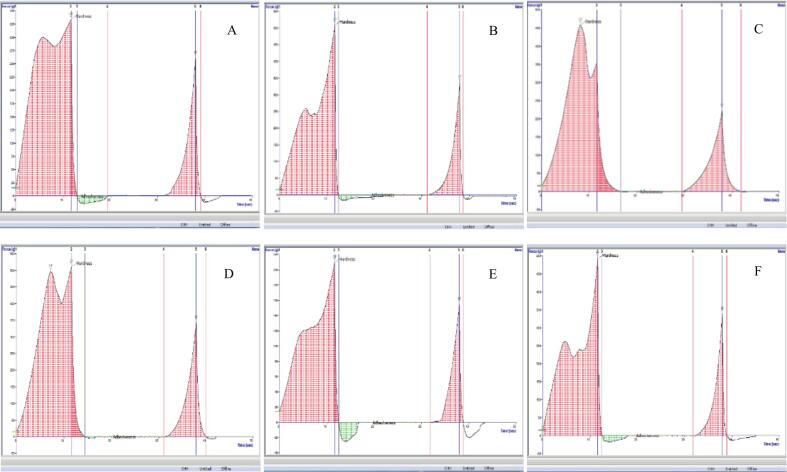
The typical graph of texture profile analysis of tofu (2x2x2 cm) prepared by adding recycled *Ficus awkeotsang* Makino seed powder and gypsum. (A): 0.2 % gypsum, 0.5 % RC-AWS; (B): 0.3 % gypsum, 0.5 % RC-AWS; (C): 0.4 % gypsum, 0.0 % RC-AWS; (D): 0.4 % gypsum, 0.5 % RC-AWS; (E): 0.4 % gypsum,1.0 % RC-AWS, and (F): 0.4 % gypsum, 1.5 % RC-AWS.

To simulate the oral mastication of tofu—a soft, semi-solid food pressed by the tongue with minimal chewing—texture profile analysis (TPA) was conducted using a 3 cm cylindrical probe compressing 2 × 2 × 2 cm tofu cubes at a speed of 1.5 mm/s. Fig. 3 illustrates the force-time curves from the first compression cycle. In the control tofu (0 % RC-AWS), the first peak—indicating structural deformation and initial rupture—exceeded the second peak, which reflected further collapse and moisture expulsion. Tofu samples containing 0.5–1.5 % RC-AWS showed a reversed pattern: the second peak exceeded the first, and the difference increased with higher RC-AWS levels— indicating greater moisture loss or structural weakness at higher concentrations. Among samples coagulated with 0.4 % calcium sulfate, hardness (defined as peak compression force) increased at 0.5 % RC-AWS addition but declined significantly at 1.0 % and 1.5 % inclusion levels (Table 3), suggesting that moderate addition strengthens the gel network. At the same time, excessive RC-AWS interferes with gel formation. Tofu containing 0.5 % RC-AWS demonstrated increasing hardness with higher coagulant concentrations (0.2 % to 0.4 %), suggesting that calcium availability enhances gel strength. Measured as the negative area during probe retraction, adhesiveness reflects the sample's stickiness. At a coagulant concentration of less than 0.4 %, the adhesiveness of tofu increased with RC-AWS levels from 0 % to 1.5 %, with the control sample exhibiting the lowest value. Among the 0.5 % RC-AWS samples, those coagulated with 0.3 % calcium sulfate had the highest adhesiveness, and 0.4 % had the lowest. This is possibly due to denser gel-limiting probe adhesion. Elasticity—measured as the ratio of deformation recovery during the second compression to the first—decreased with increasing RC-AWS content under 0.4 % coagulant, with control tofu showing the highest elasticity. Notably, elasticity declined slightly as calcium sulfate concentration increased in the 0.5 % RC-AWS tofu samples, suggesting denser gels have slower structural recovery. Cohesiveness—defined as the ratio of total energy absorbed during the second compression to that of the first—was not significantly affected by either RC-AWS or calcium sulfate levels, indicating a consistent internal bonding capacity across samples. Gumminess—a derived parameter (hardness × cohesiveness)—followed the same trend as hardness due to the minimal variation in cohesiveness. Chewiness—defined as gumminess × elasticity—showed similar patterns. Tofu with 0.5 % RC-AWS and 0.4 % coagulant had the highest chewiness, whereas samples with 1.5 % RC-AWS had the lowest values, indicating a soft and less resilient texture. Calculated as the ratio of energy absorbed before and after the peak force in the first compression, resilience demonstrates the sample's structural robustness. Resilience decreased with the addition of RC-AWS under constant coagulant conditions (0.4 %), though the differences among RC-AWS inclusion levels were not statistically significant. Calcium sulfate concentration did not significantly affect resilience in 0.5 % RC-AWS tofu. Insufficient coagulant (≤0.2 %) resulted in soft, sticky tofu, while 0.5 % RC-AWS powder improved texture by increasing hardness, elasticity, and chewiness. However, excessive addition (≥1.0 %) led to a deterioration in these properties. These observations align with past research. Abd Karim et al. (1999) and Shen and Kuo (2017) reported that polysaccharide additives (e.g., carrageenan) in protein gels influence tofu textural properties by altering gelation behavior through interactions with calcium ions and soy proteins. Given the high total carbohydrate content of RC-AWS, including potential pectin residues, it is likely that RC-AWS alters protein-polysaccharide interactions, particularly under calcium-rich conditions. When RC-AWS is added in moderation (e.g., 0.5 %), it may support network formation. However, excessive addition likely disrupts the protein gel matrix by over-cross-linking or absorbing water, weakening the tofu structure.

### 3.7 Sensory evaluation of soy Milk and tofu fortified with RC-AWS powder

#### 3.7.1 Sensory Acceptance Test for RC-AWS Soy Milk

**Fig. 4:**
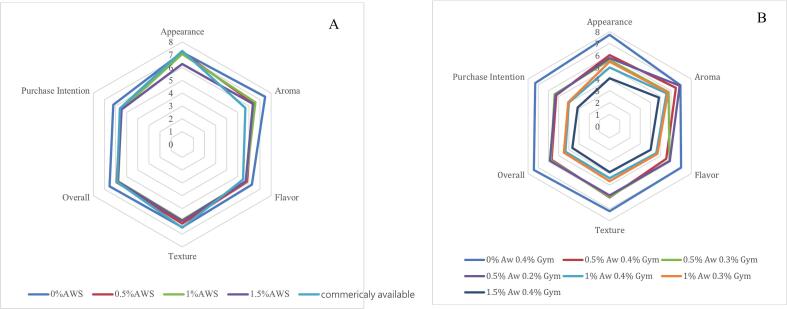
The sensory evaluation and consumer acceptance testing of soybean milk (A) and tofu (B) prepared by adding recycled *Ficus awkeotsang* Makino seed powder.

This study conducted a consumer acceptance test (*n* = 50) to evaluate soy milk fortified with 0 %, 0.5 %, 1 %, and 1.5 % RC-AWS powder. Fig. 4A shows no significant differences among the experimental samples and commercial soy milk in appearance scores, except for the 1.5 % RC-AWS soy milk, which received significantly lower ratings. Regarding aroma, the 0 % RC-AWS soy milk achieved the highest score, while commercial sugar-free soy milk received the lowest. Incorporating RC-AWS introduced a mild, grassy note, while the commercial sample contained chicory fiber and salt, potentially affecting aroma perception. Although RC-AWS soy milk was filtered during preparation, panelists still noted a gritty mouthfeel and a distinct aiyu flavor, which slightly reduced overall and texture scores. Nonetheless, all experimental samples received average scores above 5 (neutral liking) and ranked higher than commercial soy milk in preference rankings. This finding suggests that RC-AWS soy milk at inclusion levels between 0.5 % and 1.5 % was generally acceptable to consumers.

#### 3.7.2 Sensory Acceptance Test for RC-AWS Tofu

Fig. 4B illustrates the sensory acceptance scores for tofu prepared with 0–1.5 % RC-AWS powder and 0.2–0.4 % gypsum. At a fixed gypsum level of 0.4 %, the tofu without RC-AWS (0 %) scored highest in appearance, aroma, flavor, texture, overall acceptance, and purchase intention. Scores gradually declined with increasing RC-AWS powder, indicating a negative correlation between inclusion levels and sensory appeal. When the RC-AWS addition was fixed at 0.5 %, differing gypsum levels (0.2–0.4 %) only slightly affected appearance, aroma, and flavor but had no significant influence on texture, overall acceptance, or purchase intention. A 9-point hedonic scale score of 5 was used as the acceptability threshold. Results indicate that tofu with RC-AWS levels below 0.5 % was deemed acceptable. Rank tests indicate that increased RC-AWS was associated with lower preference rankings. At the same time, gypsum concentration had a minimal impact, suggesting that RC-AWS level was the primary factor influencing consumer preference.

#### 3.7.3 Descriptive Analysis of RC-AWS Tofu

**Fig. 5:**
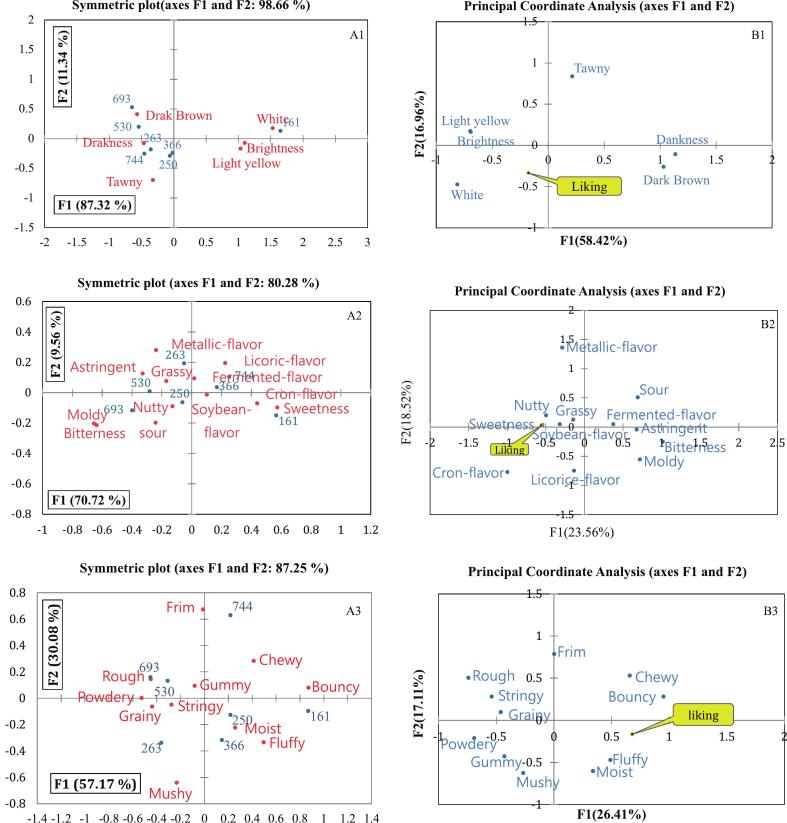
The symmetric plot (A) and principal coordinate analysis (B) in the appearance (A1, B1), flavor (A2, B2), and texture (A3, B3) descriptive analysis of tofu prepared by adding recycled *Ficus awkeotsang* Makino seed powder and gypsum. 161: 0 % RC-AWS powder and 0.4 % Gypsum;250: 0.5 % RC-AWS powder and 0.4 % Gypsum;744: 0.5 % RC-AWS powder and 0.3 % Gypsum;366: 0.5 % RC-AWS powder and 0.2 % Gypsum;530: 1 % RC-AWS powder and 0.4 % Gypsum;263: 1 % RC-AWS powder and 0.3 % Gypsum;693: 1.5 % RC-AWS powder and 0.4 % Gypsum.

Fig. 5 A1 shows the correspondence analysis (CA) biplot of tofu appearance descriptors. The first and second axes explained 87.32 % and 11.34 % of the total variance, respectively (98.66 % cumulative). All appearance attributes—except “yellow-brown”—differed significantly (*p* < 0.0001). Tofu without RC-AWS (sample 161) was characterized by “white,” “light yellow,” and “brightness.” In contrast, tofu samples containing 0.5–1.5 % RC-AWS (samples 250, 366, 744, 530, 263, and 693) were associated with “dullness” and “dark brown,” particularly samples 693 and 530. In Fig. 5 B1, which overlays liking scores with appearance descriptors, “white,” “light yellow,” and “brightness” are closely aligned with the liking centroid. This finding suggests panelists preferred tofu with a lighter appearance, consistent with findings of Ullah et al. (2019), who reported that color is a key factor in consumer acceptance, with high-quality tofu perceived as white or light yellow. Fig. 5 A2 presents the flavor CA results. The first two axes explain 70.72 % and 9.56 % of the variance, respectively (80.28 % cumulative). Tofu with 0 % RC-AWS was positioned near the “sweet” descriptor, while tofu with 0.5–1.5 % RC-AWS was aligned with “astringent” and “musty” notes. Lower gypsum levels were associated with samples closer to the “sweet” region. In the flavor–liking overlay (Fig. 5 B2), “sweet,” “beany,” “grassy,” and “nutty” were positively associated with liking, while “musty,” “bitter,” and “astringent” were negatively correlated. Thus, excessive RC-AWS or gypsum is likely to introduce undesirable flavors. Fig. 5 A3 shows the CA of textural attributes. The first and second axes explained 57.17 % and 30.08 % of the total variance, respectively (87.25 % cumulative). Tofu with 0 % and 0.5 % RC-AWS was associated with “chewy,” “springy,” and “moist” characteristics, while samples with 1 % and 1.5 % RC-AWS were linked to “grainy” and “rough” textures. Among 0.5 % RC-AWS tofu samples, 0.3 % gypsum yielded a firmer texture, and 0.2 % and 0.4 % gypsum resulted in higher moisture. The texture–liking plot (Fig. 5 B2) shows that panelists preferred tofu with “chewiness,” “springiness,” “fluffiness,” and “moistness.” Conversely, “fiber,” “roughness,” and “graininess” were linked to lower preference. These findings align with the instrumental TPA results (Table 4), indicating that 0.5 % RC-AWS enhances the hardness, elasticity, gumminess, and chewiness of tofu; higher levels (1–1.5 %) diminish its textural quality. Further, low gypsum levels result in soft tofu, while high levels create excessive firmness.

**Table 4:** The Texture profile analysis of tofu prepared by adding recycled *Ficus awkeotsang* Makino seed powder and gypsum.

Gypsum (%)	RC-AWS (%)	Hardness (g)	Adhesiveness (g.*sec*)	Springiness (mm)	Cohesivenes(s)	Gumminess (g)	Chewiness (g × mm)	Resilience
0.4	0.0	648.8 ± 323.3^b^	−35.3 ± 28.3^a^	0.71 ± 0.17^b^	0.23 ± 0.06^a^	142.5 ± 71.7^b^	106.5 ± 70.8^a^	0.07 ± 0.02^a^
	0.5	797.8 ± 359.9^a^	−35.7 ± 22.2^a^	0.69 ± 0.18^b^	0.21 ± 0.07^ab^	168.2 ± 85.2^a^	113.5 ± 62.4^a^	0.05 ± 0.01^b^
	1.0	468.5 ± 85.3^c^	−76.8 ± 42.5^b^	0.50 ± 0.13^c^	0.22 ± 0.04^ab^	102.7 ± 27.4^c^	52.0 ± 21.2^cd^	0.05 ± 0.02^b^
	1.5	431.8 ± 64.8^c^	−76.6 ± 59.9^b^	0.44 ± 0.11^c^	0.19 ± 0.05^b^	82.2 ± 23.3^cd^	37.5 ± 17.2^de^	0.04 ± 0.02^b^
0.3	0.5	446.6 ± 119.7^c^	−70.9 ± 29.2^b^	0.74 ± 0.26^b^	0.24 ± 0.11^a^	98.3 ± 21.2^cd^	75.4 ± 35.0^b^	0.06 ± 0.04^ab^
0.2	0.5	376.6 ± 95.6^c^	−50.3 ± 22.6^a^	0.90 ± 0.44^a^	0.22 ± 0.05^ab^	77.3 ± 13.5^de^	69.0 ± 34.2^bc^	0.05 ± 0.03^ab^

Data are expressed as mean ± standard deviation (n = 3).

Data with different letters within the same column are significantly different (p < 0.05).

RC-AWS: Recycled *Ficus awkeotsang* Makino seed powder.

## 4 Conclusion

The composition of residual aiyu seeds (RC-AWS) changed significantly following the traditional gel extraction process in this study. Compared to untreated seeds, RC-AWS showed reduced levels of crude fiber (pectin), crude fat, and crude protein, while showing higher proportions of total carbohydrates, ash, and iron. Among various solvent extractions, the total phenolic and flavonoid contents did not consistently align with antioxidant activity, suggesting the presence of other antioxidative constituents. Notably, although not the richest in phenolics or flavonoids, the 70 % ethanol extract demonstrated the highest antioxidant activity. This finding indicates that this solvent system may extract non-phenolic antioxidants from RC-AWS powder more effectively.

As RC-AWS powder levels increased in soymilk formulations, total solids content paradoxically decreased. This is likely due to the gelling effect of residual pectin in the powder, which may enhance coagulation and lead to greater filtration loss during preparation. Additionally, pectin and soy proteins can interact with Ca^2+^ ions, potentially affecting tofu's water-holding capacity, protein content, and textural characteristics. Thermal processing during soymilk preparation likely caused the degradation of antioxidant compounds in RC-AWS. This resulted in lower total phenolics and flavonoids in the ethanol extracts of the final product compared to direct RC-AWS extracts. Nonetheless, antioxidant content and activity in soymilk increased significantly with higher inclusion levels of RC-AWS powder. The texture of tofu is primarily determined by the strength of the protein gel. Adding RC-AWS powder at levels exceeding 0.5 % interfered with optimal soy protein coagulation, leading to softer and stickier textures. Sensory evaluations also indicate that increasing the RC-AWS powder imparted a reddish-brown color, grittiness, and a distinct herbal-like flavor, negatively affecting consumer preference. Despite these sensory changes, soymilk fortified with 0.5–1.5 % RC-AWS powder and tofu with 0.5 % RC-AWS powder were still deemed acceptable among panelists. No significant differences in overall liking were observed among the RC-AWS soymilk samples. This finding suggests that RC-AWS powder is suitable for the nutritional enhancement of soymilk—particularly for increasing fiber, iron, and antioxidant content. However, in tofu, RC-AWS powder levels above 0.5 % were less favored in this study, limiting its applicability for high-level fortification in tofu products. Thus, incorporating RC-AWS powder into soymilk and tofu is a feasible approach for enhancing nutritional value while improving the reutilization and economic value of this agricultural by-product.

## CRediT authorship contribution statement

**Shu-Hua Chiang:** Methodology, Formal analysis. **Yung-Chung Chang:** Resources, Formal analysis. **Chen-Ting Hsiao:** Software, Data curation. **Chih-Wei Chen:** Writing – review & editing, Writing – original draft, Investigation, Conceptualization.

## Declaration of competing interest

The authors declare that they have no known competing financial interests or personal relationships that could have appeared to influence the work reported in this paper.

## Data Availability

No data was used for the research described in the article.
